# Eco-anxiety and Psychosocial Problems: A Systematic Review

**DOI:** 10.1192/j.eurpsy.2025.1880

**Published:** 2025-08-26

**Authors:** M. T. Sinan, C. H. Ayhan, M. C. Aktaş, S. Aktaş, K. Aslan

**Affiliations:** 1 Mental Health And Pschiatric Nursig; 2 Departman of Psyhciatric, Van Yuzuncu Yil University, VAN, Türkiye

## Abstract

**Introduction:**

Eco-anxiety, a term that encapsulates the anxiety and distress associated with climate change and environmental degradation, has emerged as a significant psychosocial issue affecting individuals across various demographics. This phenomenon is particularly pronounced among younger generations, who often experience heightened awareness of climate-related threats and their potential impacts on future well-being. The interplay between eco-anxiety and psychosocial problems is complex, involving emotional, cognitive, and social dimensions that can significantly influence mental health outcomes. Research indicates that eco-anxiety is characterized by a future-oriented worry about the potential impacts of climate change, distinguishing it from other eco-emotions such as eco-grief and eco-despair. While many individuals experience eco-anxiety in a non-clinical form, there are instances where it can escalate to pathological levels, leading to significant mental health challenges. This is particularly relevant for young people, who may face multiple life stressors, such as academic pressures and social expectations, which can exacerbate feelings of eco-anxiety and contribute to the development of mental health issues.

**Objectives:**

In this study, the negative effects of climate change will be emphasized and its effects on human health and psychology will be emphasized. The main purpose of the study is to prepare the ground for future studies on eco-anxiety, which addresses the connection between climate change and psychology, and to increase social awareness.

**Methods:**

The study will conduct between October 2024 and January 2025 2023 in 3 databases (PubMed, Cochrane Library, Science Direct) using the keywords “eco-anxiety” “psychosocial problems” and “mental health”. These databases were preferred because they contain a significant amount of evidence-based literature in the field of biomedical sciences and psychology. Studies conducted between 2000 and 2024, whose full texts were accessed and written in Turkish and English were included in the study.

**Results:**

20 national and international research articles on the subject have been reached and the literature review continues. When the literature review is finalized, all study results will be presented together.

**Conclusions:**

Conclusions: In summary, eco-anxiety represents a significant psychosocial challenge that intertwines with various mental health issues. Understanding the emotional, cognitive, and social dimensions of eco-anxiety is crucial for developing effective interventions that address the mental health impacts of climate change. By fostering emotional regulation, acknowledging the role of grief, and promoting community engagement, mental health professionals can better support individuals grappling with eco-anxiety and its implications for overall well-being.

**Disclosure of Interest:**

None Declared

